# A Unified Multitask Architecture for Predicting Local Protein Properties

**DOI:** 10.1371/journal.pone.0032235

**Published:** 2012-03-26

**Authors:** Yanjun Qi, Merja Oja, Jason Weston, William Stafford Noble

**Affiliations:** 1 Machine Learning Department, NEC Labs America, Princeton, New Jersey, United States of America; 2 Department of Genome Sciences, University of Washington, Seattle, Washington, United States of America; 3 Google, New York, New York, United States of America; The Centre for Research and Technology, Hellas, Greece

## Abstract

A variety of functionally important protein properties, such as secondary structure, transmembrane topology and solvent accessibility, can be encoded as a labeling of amino acids. Indeed, the prediction of such properties from the primary amino acid sequence is one of the core projects of computational biology. Accordingly, a panoply of approaches have been developed for predicting such properties; however, most such approaches focus on solving a single task at a time. Motivated by recent, successful work in natural language processing, we propose to use *multitask learning* to train a single, joint model that exploits the dependencies among these various labeling tasks. We describe a deep neural network architecture that, given a protein sequence, outputs a host of predicted local properties, including secondary structure, solvent accessibility, transmembrane topology, signal peptides and DNA-binding residues. The network is trained jointly on all these tasks in a supervised fashion, augmented with a novel form of semi-supervised learning in which the model is trained to distinguish between local patterns from natural and synthetic protein sequences. The task-independent architecture of the network obviates the need for task-specific feature engineering. We demonstrate that, for all of the tasks that we considered, our approach leads to statistically significant improvements in performance, relative to a single task neural network approach, and that the resulting model achieves state-of-the-art performance.

## Introduction

Proteins participate in every major biological process within every living cell. Therefore, elucidating protein function is a central endeavor of molecular biology. In this work, we focus on predicting local functional properties, which can be summarized as a labeling of amino acids. Many important functional properties can be described in this fashion, including secondary structure, solvent accessibility, transmembrane topology and the locations of signal peptides, DNA-binding residues, protein-binding residues and coiled-coil regions.

Our work is motivated, in part, by recent, successful work in the field of natural language processing [1]. Analogous to functional labeling of amino acids, natural language can be annotated with tags indicating synonymous pairs of words, parts of speech, larger syntactic entities, named entities, semantic roles, etc. These labelings exhibit strong dependencies across tasks. Accordingly, Collobert and Weston [1] demonstrated that a unified neural network architecture, trained simultaneously on a collection of related tasks, provides more accurate labelings than a network trained only on a single task. Their study thus demonstrates the power of *multitask learning*, which has been the subject of much recent work in machine learning [2]. Furthermore, essential to the success of the Collobert and Weston system is the use of a *deep neural network* [3], [4], which is able to learn a hierarchy of features that are relevant to the tasks at hand given very basic inputs. The deep network employs different layers to represent cross-task and task-specific information. Thus, the deep multitask architecture makes it possible to avoid the challenging process of incorporating hand-engineered features specific to each task. Finally, a critical piece of the Collobert and Weston methodology is the use of a so-called *language modeling* task [1], in which the network learns to discriminate between genuine natural language sentences and synthetically generated sentences. Our work makes use of all three of these components–multitask learning, deep learning and an analog of the language model–to predict local protein properties.

Currently, most approaches to predict local protein properties focus on one task at a time. Perhaps the most well-studied such problems are the canonical secondary structure prediction problem [5] and the related task of predicting transmembrane protein topology [6]. Other tasks include identifying signal peptides, predicting DNA-binding residues, identifying coiled-coil regions, predicting relative or absolute solvent accessibility, etc. However, like the natural language processing tasks mentioned above, all of these protein labeling tasks exhibit strong inter-task dependencies. For example, transmembrane protein topology and secondary structure prediction are closely linked, because most transmembrane protein segments are alpha helices. Similarly, signal peptide prediction can be viewed as prediction of a particular type of transmembrane segment. DNA-binding and protein-binding residues may also share similar structural features, since both must be exposed on the surface of the protein. Some work makes use of these dependencies in a pipelined fashion; for example, [7] use secondary structure and solvent accessibility predictions as input to a DNA-binding residue predictor. A drawback to the pipelining approach is that errors from one classifier get propagated to downstream classifiers [8]. A more elegant and robust approach is to use only the primary amino acid inputs, combine the prediction problems, and learn the tasks simultaneously using multitask learning. This strategy also avoids extracting task-specific sets of features.

In this work, we define a unified architecture for prediction of local protein properties by training a deep neural network in a multitask fashion. Specifically, our network learns simultaneously to predict solvent accessibility, transmembrane topology and two secondary structure alphabets, and to identify DNA-binding residues, protein-binding residues, signal peptides and coiled-coil regions. We also include a semi-supervised learning task, which we call *natural protein modeling*, to learn features representing local amino acid patterns in naturally occurring protein sequences. We evaluate this architecture using benchmark datasets for each task. The results show that multitask learning improves performance on nearly every task, and adding the semi-supervised natural task helps in nearly every case. Furthermore, adding both multitask learning and the natural protein task makes our architecture achieve state-of-art performance on almost all tasks.

## Methods

### Prediction tasks

A variety of local protein properties can be represented as predicting labels of amino acids. In this work, we predict ten such labelings, each of which is described below.

#### Secondary structure

A protein's *secondary structure* is a useful intermediate between the relatively easy-to-ascertain primary amino acid sequence and the difficult-to-obtain three-dimensional structure. The secondary structure specifies the general three-dimensional form of local segments of a protein. The most commonly observed local structures are 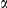-helices, 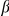-sheets and loops. In the secondary structure prediction task we aim to predict each residue's secondary structure label. Knowing a protein's secondary structure may yield insight into the protein's functional class, suggest boundaries between domains, or aid in inferring the protein's 3D structure.

Since the advent of the first automated secondary structure prediction method 22 years ago [5], dozens of subsequent methods have been described in the scientific literature. These include methods that employ neural networks [5], [9]–[13] and probability models such as hidden Markov models [14] and dynamic Bayesian networks [15].

Our multitask learning data set includes three protein secondary structure prediction tasks. The first is a standard benchmark, CB513 [16], consisting of 513 unrelated proteins with known 3D structure. To create the other two secondary structure prediction tasks, we used 11 795 chains from the DSSP [17]. We considered two variants of the secondary structure prediction task, one task using the full 8-letter alphabet and one task using the reduced, 3-letter alphabet.

From the CB513 benchmark we eliminated 16 proteins because they were shorter than 30 amino acids. The CB513 data is labeled with the 8-letter DSSP alphabet (H = alpha helix, B = residue in isolated beta bridge, E = extended strand, G = 3-helix, I = 5-helix, T = hydrogen bonded turn, S = bend, L = loop) [17]; however, for comparison with other methods that use this benchmark, we reduce the 8-letter alphabet to a 3-letter alphabet in the standard way [9]: 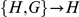, 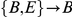, and 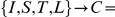
Coil.

To create the other two secondary structure prediction tasks, we used the DSSP, downloaded on Feb 1, 2008. After removing short sequences and sequences comprised primarily of Xs, and after filtering so that no pair of sequences shares 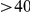
% sequence identity, we were left with 11 795 protein chains. We considered two variants of the secondary structure prediction task, one task using the full 8-letter alphabet and one task using the reduced, 3-letter alphabet.

Note that we cite the best previously reported accuracy (i.e., the highest percentage of correct predictions) on CB513 as 80.0% [18]. There is actually a recently published paper that reports 80.49% accuracy [19]; however, in corresponding with the authors of that paper, we learned that this value is not based on a cross-validated test.

#### Transmembrane topology and signal peptide prediction

Complementary to prediction of protein secondary structure is the prediction of transmembrane topology. The most common type of transmembrane protein consists of a series of 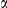-helices that span the membrane, interleaved with loops that extend out of the membrane. The labeling task consists of identifying these membrane-spanning segments and then specifying whether each loop is inside or outside of the membrane; hence, transmembrane predictors employ a three-letter alphabet.

Transmembrane proteins are of particular interest for two reasons. First, because transmembrane proteins cannot be crystallized (due to the presence of the membrane), their 3D structure cannot be easily determined. Second, because of their importance in communicating across membranes, more than half of all drug targets are transmembrane proteins, even though only an estimated 18–26% of all proteins are transmembrane proteins [20]–[22].

A closely related task to the prediction of transmembrane protein topology is the prediction of signal peptides, which are short (3–60 amino acids) peptides that direct newly translated proteins to their final destinations in the cell. Early methods predicted signal peptides [23], [24] and transmembrane protein topology separately [6]. More recent work suggests that the two tasks can be solved more effectively using a joint predictor [25], [26].

For the signal peptide and transmembrane topology prediction tasks, we use data from three sources. First, we use 1087 globular proteins described in [25]. These proteins are all labeled completely with “O's”, corresponding to outside (non-cytoplasmic) loops. Second, we combined the transmembrane proteins from [25] and [27], resulting in a nonredundant set of 46 membrane proteins with signal peptides and 324 membrane proteins without signal peptides. These proteins are labeled with a five-letter alphabet: S = signal peptide, O = outside loop, I = inside loop, M = membrane-spanning alpha helix and R = re-entrant region. Some residues are unlabeled (indicated with “.”). Third, we use 1729 signal peptide proteins from [28]. In each of these proteins, the signal peptide is labeled “S”, and the rest is unlabeled (We use “N” to indicate non-signal peptide regions). Using these three data sets, we consider two tasks: the full, five-letter SP+TM topology prediction task on the first two data sets, as well as the three-letter signal peptide detection task using the first and third data sets.

For validation on both tasks, we use the 10-fold cross-validation splits from [26]. For the transmembrane topology problem, a predicted transmembrane segment is deemed correct if it overlaps a true transmembrane segment by at least five amino acids, whereas inside, outside and signal peptide predicted segments require only a single amino acid overlap. The figure of merit is the segment level metric for the transmembrane prediction task, including both sensitivity and precision. This metric is only computed with respect to the 324 TM or 46 TM+SP proteins in the data. For signal peptide prediction, the figure of merit is protein level accuracy, where a protein is deemed to have a predicted signal peptide if any residue therein is assigned the label “S.”

#### Solvent accessibility

The solvent accessibility prediction task involves distinguishing between amino acids that are accessible to water versus amino acids that are buried inside the protein. Hence, the task involves a two-letter alphabet. Defining this alphabet requires setting a threshold, which can be done either on an absolute scale or relative to the protein in which the amino acid resides.

Early methods for solvent accessibility prediction used neural networks [10], [29], [30] or support vector machines [31]. [32] showed that a simple baseline predictor performs rather well relative to more sophisticated methods, and [33] made a consensus predictor by combining predictions from three different methods.

We used the DSSP to define two solvent accessibility data sets. The DSSP reports the solvent accessibility of each amino acid on an absolute scale (surface area accessible to water in units of Å^2^). Therefore, we first defined a binary alphabet using a threshold of 15. Second, we computed the relative accessibility of each amino acid by dividing each accessibility value by the per-protein maximum, and we defined a second binary labeling using a threshold of 0.15 [34]. We computed these two labelings across the same collection of 11 795 sequences that were used for the secondary structure task.

#### Coiled coil regions

A coiled coil is a protein structural motif, in which 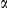-helices are coiled together like the strands of a rope. Coiled coils usually contain a repeated pattern, hpphppp, of hydrophobic (h) and polar (p) amino-acid residues, referred to as a heptad repeat. The positions in the heptad repeat are usually labeled abcdefg, where a and d are the hydrophobic positions, often being occupied by isoleucine, leucine or valine. Folding a sequence with this repeating pattern into an alpha-helical secondary structure causes the hydrophobic residues to be presented as a “stripe” that coils gently around the helix in left-handed fashion, forming an amphipathic structure. Coiled coil domains function in the stabilization of tertriary and quaternary structure of proteins. Many coiled coil proteins are involved in protein-protein interactions and have important biological functions, such as protein trafficking, signalling and regulation of gene expression.

The first method for predicting coiled coil regions used position specific scoring matrices to score sequence windows [35]. Subsequent methods achieved improved accuracy by including correlations among residues [36]–[38] or by using hidden Markov models [39]. Currently, the best performing coiled coil predictor is an HMM that uses evolutionary information [40].

For the coiled coil prediction task, we downloaded a dataset used in the training of the Paircoil [38] algorithm from the PPT-DB [41] database server. The data set contains 776 proteins. In each of these proteins, consecutive 7-residue stretches in the coiled coil region are labeled with the sequence “abcdefg”. The most frequent label, at 69.8%, is “N”, the label for non-coiled residues. Each of the other labels has a frequency of about 4.3%.

We evaluate the performance of our method by using the ten-fold cross-validation, as in the PPT-DB [41] database server. We evaluate this task using both the amino acid level accuracy and the “Percent correct” metric proposed in [41]. The latter is computed as the percentage of matching structure regions where matches are any aligned coiled-coil segments (e.g. “a” matches “c”), and “N” matching “N”; otherwise, a mismatch is counted.

#### DNA binding

The final two prediction tasks involve identifying amino acids that interact with, respectively, DNA molecules or other proteins. Detection of DNA-binding sites in proteins is critical for targeting gene regulation and manipulation. Thousands of proteins are known to bind to DNA; however, for most of these proteins the mechanism of action and the residues that bind to DNA, i.e. the binding sites, are not known. Experimental identification of binding sites requires expensive and laborious methods such as mutagenesis and binding assays. If the 3D structure of a protein is known, then it is often possible to predict DNA-binding sites *in silico*. However, for most proteins, such knowledge is not available.

Several methods have been developed to predict DNA-binding residues from the primary amino acid sequence. [42] described predictors based on sequence composition and predicted solvent accessibility. Later, some of the same authors [43] used profiles of homologous proteins to achieve more accurate prediction of DNA-binding sites. More recently, [7] used three types of inputs–PSI-BLAST profiles, predicted secondary structure, and predicted solvent accessibility–to train a support vector machine DNA-binding predictor.

For the prediction of DNA binding residues, we use a data set of DNA/protein structures collected by [7]. The set contains 693 DNA-binding protein sequences with an average length of 183 amino acids. Residues are considered DNA-binding if they are 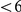 Å from the DNA molecule. The label alphabet consists of two characters (B = binding, N = not binding), and 18.8% of the amino acids in this set are labeled “B.”

To evaluate performance on this data set, we use a clustering of the protein chains such that no inter-cluster pairs are too similar. This is the same clustering that was used by [7]. Their HSSP threshold of zero [44], [45] corresponds to 20% pairwise sequence identity. We then perform three-fold cross-validation on the clusters, i.e., we randomly divide the set of clusters into three equal-sized portions, and we repeatedly train on two-thirds and test on the remaining one-third of the data.

#### Protein binding residues

The protein-binding prediction task is analogous to the prediction of DNA-binding residues, but focused on binding sites for protein-protein interactions rather than protein-DNA interactions. Identifying these sites from the primary amino acid sequence is critical to understanding protein function, because so many proteins carry out their functions as part of multi-protein complexes.

Several studies attempted to address the sequence-based interaction site prediction problem. Pazos et al. [46] use multiple sequence alignment to detect correlated changes in a group of interacting protein domains for predicting contacting pairs of protein residues. Gallet et al. [47] analyze hydrophobicity patterns and amino acid distributions in known interaction sites to identify linear stretches of sequences. Yan et al. [48] apply support vector machines to predict protein binding sites with features extracted from sequence neighbors for each target residue. Liang et al. [49] predict interface residues using an empirical score function that is a linear combination of the energy score, interface propensity and residue conservation score. And Ofran et al. [50] employ a neural network approach using PSI-BLAST profile features to identify interaction sites directly from sequences.

For the prediction of protein binding residues, we use a data set obtained from [50]. The authors used non-redundant subsets from PDB, focusing on transient interactions between two non-identical chains of two different proteins. This approach yielded 1133 proteins. A residue was defined to be in a protein-protein interaction if any of its atoms was 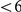 Å from any atom in the other protein. The three-fold cross validation splits were also obtained from [50], where sequence-unique subsets were built for all types of proteins under consideration.

### Deep neural network for each task

**Figure 1:**
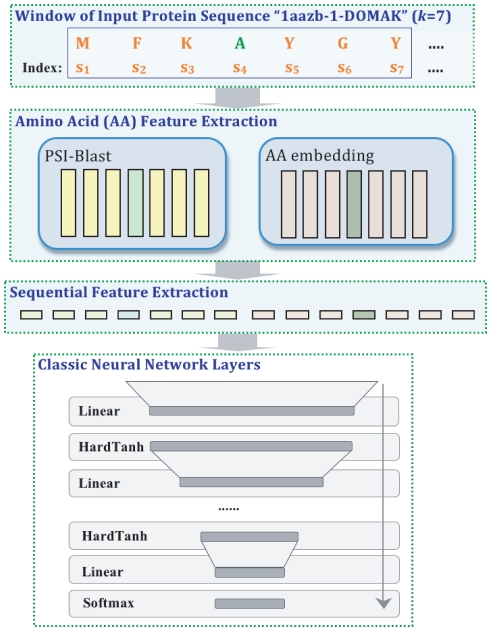
Deep neural network architecture. Given an input amino acid sequence, the neural network outputs a posterior distribution over the class labels for that amino acid. This general deep network architecture is suitable for all of our prediction tasks. The network is characterized by three parts: (1) an amino acid feature extraction layer, (2) a sequential feature extraction layer, and (3) a series of classical neural network layers. The first layer consists a PSI-BLAST feature module and an amino acid embedding module. With a sliding window input 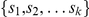 (here 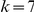), the amino acid embedding module outputs a series of real valued vectors 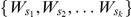. Similarly, the PSI-BLAST module derives 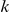 20-dimensional PSI-BLAST feature vectors corresponding to the 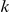 amino acids. These vectors are then concatenated in the sequential extraction layer of the network. Finally, the derived vector is fed into the classical neural network layers. The final softmax layer allows us to interpret the outputs as probabilities for each class.

We introduce a deep neural network architecture for protein labeling tasks. The input sequence is fed through several layers of feature extraction, and features relevant to the task are learned automatically by backpropagation in deep layers of the network. The general deep network architecture, which is suitable for all our prediction tasks, is summarized in Figure 1. The network is characterized by two specialized layers–an amino acid feature extraction layer and a sequential feature extraction layer–followed by a series of classical neural network layers.

#### Amino acid feature extraction

The first layer extracts features for each amino acid by mapping amino acids to real-valued vectors. We use two distinct types of features–a learned embedding into a 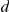-dimensional feature space, and a 20-dimensional feature representation produced by PSI-BLAST [51]–which are concatenated to produce the output of the layer.

The first feature extraction module projects each amino acid into a 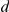-dimensional feature space, where 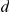 is a hyperparameter; i.e., 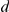 is not subject to optimization. Within a finite amino acid dictionary 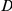, each amino acid 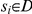 is embedded into the feature space using a 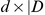 lookup table 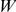, such that 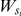, the column vector of 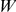 at the index of 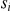, is the vector corresponding to amino acid 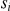. Thus, in the first layer of our architecture an input sequence 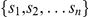 is transformed into a series of real valued vectors 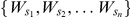. The parameters of the lookup table 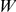 are learned automatically as part of the neural network training. This type of feature extraction–called a “local encoding of amino acids”–was originally proposed by Riis et al. [52]. The encoding weights 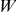 are randomly initialized with a centered, uniform distribution [53] and then learned by back-propagation. The resulting encoding is optimal in the sense that it optimizes the objective cost on the training set for the specific network and the specific task.

The second feature extraction module in the first layer extracts information from an alignment of homologous proteins identified by the PSI-BLAST algorithm. Each length-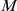 query sequence is searched using PSI-BLAST against the NCBI nonredundant protein sequence database, yielding a 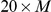 position-specific scoring matrix. Note that PSI-BLAST will create a PSSM even when no homologs are present. In this case, each column is simply a value from the specified BLOSUM matrix. Each element in the matrix represents the log-likelihood of a particular residue substitution at that position in the template. The profile matrix elements (typically in the range 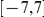) are scaled to the required range 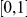 by using the following scaling function [54]:
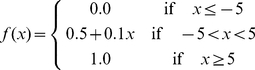
(1)where 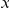 is the value from profile matrix. Each column of the rescaled matrix comprises a 20-dimensional PSI-BLAST feature vector for the corresponding amino acid.

#### Sequential feature extraction layer

To facilitate identification of local sequence structure, the second layer performs a sliding window operation on the sequence. As illustrated in Figure 1, the second layer aggregates the output of the first layer into blocks corresponding to a fixed window size 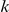. Figure 1 shows an example using a window size of 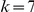, so each block contains information about the current amino acid as well as the three flanking amino acids on either side. Altogether, because the output of the first layer has 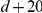 dimensions, the output of the second layer has 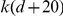 dimensions.

#### Classical neural network layers

The remaining layers comprise a standard, fully connected multi-layer perceptron network with 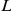 layers of hidden units. Each hidden layer learns to map its input to a hidden feature space, and the last output layer then learns the mapping from the hidden space to the output class label space. In Figure 1, the sequential feature extraction layer induces a large effective feature space, where examples correspond to each possible length-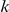 sequence of amino acids. Thus, the job of the hidden layers is to map this high dimensional input space to a lower dimensional feature space and then to look for hyperplanes that separate examples with different amino acid labels in the output layer.

In practice, the output of the 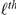 layer 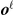, which contains 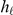 hidden units, is computed with
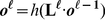
(2)where the matrix of parameters 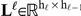 is trained by backpropagation. The transfer function 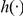 is defined as:
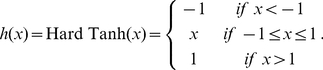
(3)The size of the last (parametric linear) layer's output 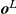 is the number of classes considered in the prediction task. This layer is followed by a softmax layer which ensures that the outputs are positive and sum to 1, allowing us to interpret the outputs of the neural network as probabilities for each class. The 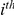 output is given by
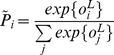
(4)In this work, for simplicity, we restrict the classical part of our neural network to one single hidden linear layer and one output linear layer.

Assuming that we are given a set of training examples 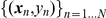, where 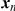 represents a local short window of amino acids and 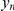 a label, the whole network is then trained to minimize the negative log-likelihood, i.e. 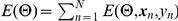 over the data with respect to 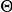: all parameters of the network. Specifically,
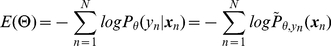
(5)Stochastic gradient descent optimization is used for the above training. Random examples 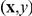 are sampled from the training set and then a gradient descent step is applied to update network parameter 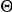 as follows:
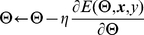
(6)where 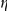 is a learning rate parameter.

### Multitasking with weight sharing of deep neural networks

**Figure 2:**
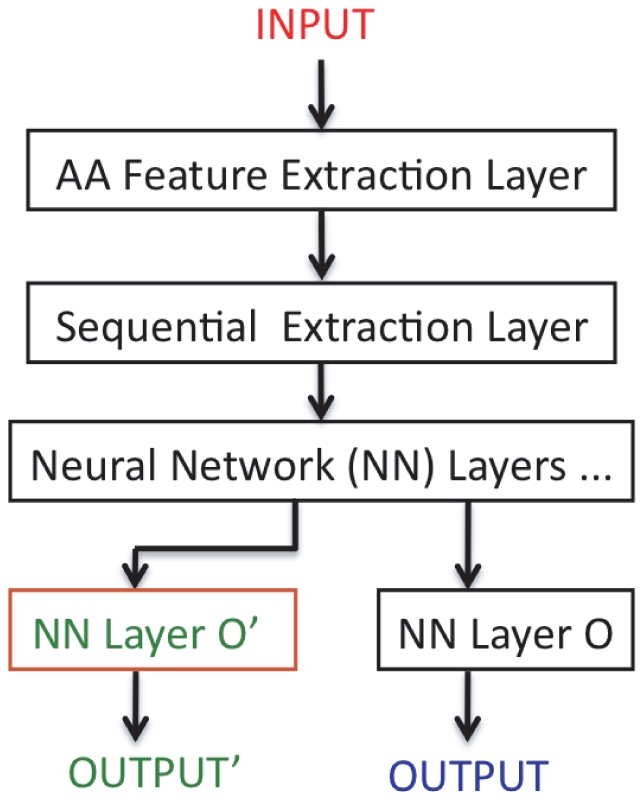
Multitask learning with weight sharing between multiple deep neural networks. In this figure, two related tasks are trained simultaneously using the network the architecture from Figure 1. Here only the very last layers of the network are task specific.

The neural network architecture displayed in Figure 1 can be adapted in various ways to perform multitask learning. In this work, we used the multitask architecture shown in Figure 2, in which the top-most layers of the network are shared across multiple tasks, and only the very last layers of the network are task specific.

Assuming we have 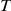 related tasks, the “weight sharing” strategy implies that the parameters for the top-most layers of the network are shared between tasks; i.e., the network includes parameters
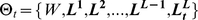
(7)for each task 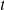. With this setup–i.e., only the last layer 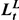 is task-specific–the neural network automatically learns an embedding that generalizes across tasks in the first layers of the network, and learns features specific for the desired tasks in the deep layers of the network.

Training in the multitask setting is accomplished by minimizing an objective function that is the sum of the objectives from each task, where each task is given equal weight. That is, we optimize:
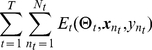
(8)assuming each task has a training set 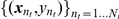. The tasks share a common feature input, and the weight sharing among 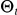 makes the optimization of different tasks dependent. When training by stochastic gradient descent, this amounts to interleaving the stochastic updates for each of the related tasks. That is, the procedure iteratively carries out the following three steps:

1. select a task at random,
2. select a random training example for this task, and
3. compute the gradients of the neural network attributed to this task with respect to this example and update the parameters.

Because some of the parameters are shared between the tasks, the tasks influence each other during training. The training procedure continues until the per-residue error becomes stable–i.e., error decreases by less than 0.00005–on a held-out validation set (one fifth of each training set was held out for this purpose) or reaches a specified maximum number of iterations. In practice, we found that 100–150 iterations are sufficient for the networks trained here.

It is worth noting that labeled data for training each task can come from completely different data sets. However, when the sizes of the training sets for the different tasks are very different, then the above procedure does not work well because the network does not train enough on the “larger” tasks. To address this problem, we employ a pre-training strategy, where the larger tasks (i.e. four large task including “ss”, “dssp”, “saa” and “sar” in Table 1) are trained jointly prior to multitasking of all tasks. This pre-training procedure ensures that the large tasks reach a stable area of the parameter space before the full multitasking, which involves all tasks. As pointed out by [55], the pre-training guides provide a regularization effect.

**Table 1:** Summary of data sets.

Name	Task	Prot Num	AA Num	CV	Composition (%)
ss	Secondary structure	11 765	2 518 596	5	41.7 = C, 21.6 = E, 36.7 = H
cb513ss	Secondary structure	497	83 707	7	42.8 = C, 22.7 = E, 34.5 = H
dssp	Secondary structure, DSSP	11 765	2 518 596	5	33.3 = H,20.4 = E, 20.1 = L,
					11.2 = T, 9.5 = S, 3.5 = G, 1.1 = B, 0.02 = I
sar	Relative solvent accessibility	11 765	2 518 596	5	51.1 = B, 48.9 = A
saa	Absolute solvent accessibility	11 795	2 518 596	5	64.9 = B, 35.1 = A
dna	DNA binding	693	127 064	3	81.2 = N, 18.8 = B
sp	Signal peptide	2816	1 058 598	10	30.8 = O, 4.0 = S, 65.2 = N
tm	Transmembrane topology	1457	460 780	10	82.1 = O, 9.6 = I, 7.5 = M,
					0.3 = S, 0.1 = R, 0.4 = N
cc	Coiled coil	765	444 138	10	69.8 = N, 4.3 = each of a/b/c/d/e/f/g
ppi	Protein protein interaction	1129	188 676	3	73.4 = P 26.6 = N

For each data set, we list the number of protein sequences, the number of amino acids, the number of cross validation folds, and the proportion of amino acids assigned to each label.

### The natural protein task: feature learning with unlabeled protein sequences

Labeling a data set can be expensive, especially when doing so requires expensive and time-consuming laboratory experiments. Consequently, the ability to leverage unlabeled data to improve a predictive model is a compelling goal. We now present a semi-supervised task to model the local patterns of amino acid contexts that occur in natural protein sequences.

This “natural protein” task is motivated by results from the natural language processing community. In that context, researchers noticed that for part-of-speech or other semantic tagging tasks, words that are semantically similar can often be exchanged with no impact on the labeling. For example, in a sentence like “the cat sat on the mat” one can replace “cat” with nouns such as “dog”, “man” or “patient” with no change in the part-of-speech tagging. Collobert and Weston [1] therefore included in their multitask learning system a task that forces two sentences with the same semantic labels to have similar representations in the shared layers of neural network, and vice versa. Training for this task is achieved by assigning a positive label to genuine fragments of natural language, and negative labels to fragments that have been synthetically generated. Essentially, this task involves learning to predict whether the given text sequence exists naturally in the English languague.

**Figure 3:**
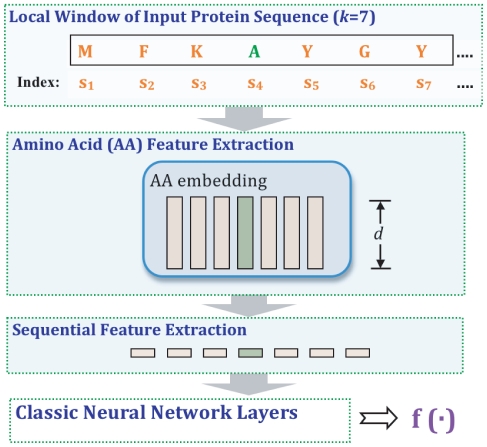
Network architecture for training the “natural protein” auxiliary task. The “natural protein” auxiliary task aiming to model the local patterns of amino acids that naturally occur in protein sequences. Using local windows in the unlabeled protein sequences as positive examples and randomly modified windows as negative examples, the network learns the feature representations for each amino acid. In contrast to the network illustrated in Figure 1, the network contains only the amino acid embedding module in the first layer of the network. The learned embedding is encoded into the real valued parameter matrix of the amino acid feature extraction layer.

Motivated by this language model, we propose an auxiliary task aiming to model the local patterns of amino acids that naturally occur in protein sequences. This is achieved by learning to predict whether the given protein segment exists in real protein sequences. Accordingly, all length-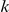 windows from SwissProt version 54.7 are labeled as positive examples, and negative fragments are generated by randomly substituting the middle amino acid in each window. Because the training set for this task is extremely large, we train the natural protein modelling task separately from the other tasks. Also, the network architecture used in this task is slightly different from that shown in Figure 1; here we do not use the PSI-BLAST feature encodings (see Figure 3). Other components of the network are the same and explained in the previous sections. As for the other tasks, the amino acid embeddings and the parameters of the subsequent neural network layers are all automatically trained by backpropagation. The difference is that here the model is trained with a ranking-type cost (with margin):
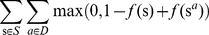
(9)where 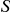 is the set of windows of amino acid segments, 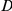 is the vocabulary of amino acids, 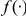 represents the output of the neural network architecture, and 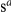 is a window where the middle amino acid has been replaced by a random amino acid 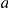. Essentially, we are learning the network weights to rank positive protein segments above synthetic segments. The training is carried out using stochastic gradient descent, which samples the cost online with respect to 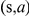. As in the natural language setting, the end goal for this training procedure is not the solution to the classification task itself, but the embedding of amino acids into a semantically meaningful, 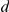-dimensional space. The real-valued vectors representing the amino acids comprise the columns of the lookup table 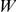 in the amino acid feature extraction layer of the network. Thus, to combine the natural protein task with other tasks, we initialize the amino acid embedding lookup table 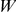 in the feature extraction layer (Figure 1) with the embedding weights learned during training of the natural protein task.

The natural protein modeling task aims to learn features representing local amino acid patterns in naturally occurring protein sequences. Conserved in natural protein sequences, these patterns are different from patterns in random sequences constructed from amino acid letters. This task is closely related to the “language model” in natural language processing, whereby language modeling aims to learn the joint probability function of sequences of words. The auxiliary task used to identify these patterns is essentially a pseudo-classification task which needs both “real” protein segments and “unreal” segments of amino acids. The synthetic negative set provides the negative segments required for this classification task.

## Results

### Data Sets

The collection of data sets tested in this paper is summarized in Table 1 and is publicly available at http://noble.gs.washington.edu/proj/multitask, along with software implementing our multitask learning strategy. The software and methods were implemented using the Torch5 (http://torch5.sourceforge.net/) machine learning library. Torch is implemented in C, and the scripting for this project was carried out in the Lua (http://www.lua.org) scripting language. The data set includes three protein secondary structure prediction tasks. The first is a standard benchmark, CB513 [16], consisting of 513 unrelated proteins with known 3D structure. To create the other two secondary structure prediction tasks, we used 11 795 chains from the DSSP database [17] downloaded on February 1, 2008. We also considered two variants of the secondary structure prediction task, one task using the full 8-letter alphabet and one task using the reduced, 3-letter alphabet. For the signal peptide (SP) and transmembrane (TM) topology prediction tasks, we define two tasks: a five-letter SP+TM topology prediction task, as well as a signal peptide detection task. We used the DSSP to define two solvent accessibility data sets, absolute accessibility and relative accessibility, in which the accessibility is scaled relative to the maximum per-protein accessibility. For the coiled coil prediction task, we use a previously described data set containing 776 proteins [38]. For the prediction of DNA binding residues, we use a data set from [7], consisting of 693 DNA-binding proteins.

### Experimental Setup

This work is predicated on a three-fold hypothesis, namely, that we can improve our ability to predict various protein labeling tasks by (1) learning an amino acid embedding, (2) using multitask learning and (3) including the “natural protein” task in our multitask learning. Accordingly, we systematically tested variants of our learning approach, with the goal of testing each of these hypotheses.

To evaluate the performance of a given predictor, we primarily focus on *accuracy* evaluated at the amino acid level, sometimes referred to as the “Q-score.” For a multiclass classifier, when we compare a predicted labeling with a true labeling of a set of proteins, each amino acid falls into one of the two categories: either the predicted label and the true label agree and the amino acid is “correct” or the predicted label and the true label disagree and the amino acid is “incorrect.” Accuracy is defined as the percentage of amino acids whose labels are predicted correctly; i.e.,
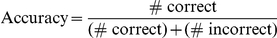
Considering the multiclass nature of the selected tasks, we also compute the precision, recall and F1 metrics separately for each class, where we treat the selected class as the positive class and all of the other classes as negatives. This essentially treats each multiclass task as separate binary classification tasks and computes separate metrics for each one. Precision for a certain class refers to the number of true positives (TP) (i.e. the number of amino acids correctly labeled as the specific class) divided by the total number of amino acids labeled as belonging to the class (i.e. the sum of true positives (TP) and false positives (FP)).
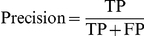
“Recall” for a certain class is defined as the number of true positives (TP) divided by the total number of amino acids that actually belong to the class (i.e. the sum of true positives (TP) and false negatives (FN)).
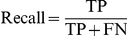
Precision and recall can be usefully combined into a single measure such as F1, defined as
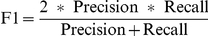
Accordingly, Table S1 summarizes the results from this calculation.

In addition, we also report the protein-level accuracy for the “sp” task, segment-level accuracy for the “tm” task, and segment-level accuracy for the “cc” task. These additional metrics allow us to compare our results to those of previously published methods.

Our learning framework requires the specification of a variety of hyperparameters. These include the size 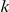 of the sequence window, the size 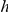 of the hidden layer, and the learning rate 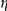. We considered 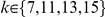, 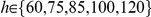, and 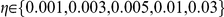. For the size of the embedding vector 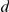 in the general embedding layer case or in the natural protein task, we tried 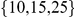. For the natural protein task, the unlabeled corpus was split into one training (70%) and one validation set. The best parameters found for the natural protein task were 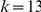, 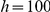 and 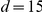. For the other tasks when training separately, we optimized each task's performance through a grid search of parameter combinations. When training jointly on multiple tasks, a grid search on parameters was also performed for the performance optimization (on average). In general, the learning rate 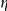 does not vary much across different tasks: for instance, the best learning rates for the tasks dssp, ssp, sar, saa, cc, sp, dna (using the abbreviations from Table 1) are all roughly 0.005. The window size 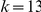 and the embedding size 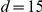 gave overall best results. Not surprisingly, the optimal number of hidden units differs depending on the inputs. For instance, when using only the PSI-BLAST feature representation for amino acids, 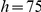 roughly performs the best for all tasks. After adding the amino acid embedding to the feature extraction layer, 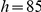 works better. Then, when training a joint model for multitasking several tasks, we found that 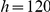 gives good performance overall.

### A learned amino acid embedding

We begin by evaluating the utility of including an amino acid embedding into the amino acid feature extraction layer of the network. The first two columns of Table 2 provide direct evidence for the utility of the learned embedding. These columns compare the performance of single-task neural networks trained using only the PSI-BLAST embedding or trained using a combination of the PSI-BLAST and learned amino acid embeddings. Considering only the amino acid-level accuracy, including the embedding improves the network's performance on 7 out of 10 tasks. Furthermore, when we consider adding the amino acid embedding in the context of multitask learning (comparing columns “Multi” and “Multi-Embed”), we observe an improvement in 9 out of 10 tasks. The largest improvement is observed for relative solvent accessibility prediction, which improves by 1.8% (from 79.2% to 81.0%) in the multitask setting.

**Table 2:** Comparison of learning strategies based on percent accuracy.

Embedding?		✓		✓	*	*	*				
Multitask?			✓	✓			✓				
Natural protein?					✓	✓	✓				
Task (%)	Single	Embed	Multi	Multi-Emb	NP	NP only	All3	All3+Vit	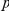 -value	CV	Previous
ss	79.1	79.6	80.5	81.3	79.7	67.7	**81.7**	81.4	1e-4	100	–
cb513ss	76.1	74.5	79.8	80.2	74.8	65.8	80.2	**80.3**	1e-3	100	80.0 [18]
dssp	65.5	66.3	67.1	68.1	66.3	54.3	68.2	**68.2**	1e-4	100	–
sar	78.4	79.8	79.2	81.0	79.8	73.1	81.0	**81.1**	1e-4	100	–
saa	80.7	81.3	81.7	82.6	81.3	74.2	**82.6**	**82.6**	1e-4	100	–
dna	82.4	82.2	85.3	87.0	82.3	81.1	88.6	**89.2**	1e-4	66.7	89.0 [7]
sp	80.9	80.7	83.6	83.9	80.7	69.4	84.1	**91.0**	1e-4	100	–
sp (prot)	99.5	99.5	99.8	99.8	99.8	99.8	99.7	99.8	5e-2	–	97.0 [26]
tm	87.1	87.5	89.0	89.3	87.7	85.8	89.4	**92.1**	1e-4	100	–
tm (seg)	91.0	96.9	97.4	98.3	96.7	92.7	**98.4**	96.5	1e-4	–	94.0 [26]
cc	88.6	89.9	93.1	94.2	90.7	87.3	94.4	**96.6**	1e-4	100	–
cc (seg)	90.7	91.9	94.5	95.6	92.0	89.7	95.7	**97.4**	1e-4	–	94.0 [41]
ppi	73.6	73.6	**78.4**	73.1	73.6	71.0	74.3	75.6	1e-4	66.7	– [50]

The table lists, for each prediction task, the per-residue percent accuracy achieved via single-task training of the neural network with just the PSI-BLAST features (“Single”), single-task training that includes the amino acid embedding (“Embed”), multitask training just using the PSI-BLAST features (“Multi”), multitask training including the amino acid embedding (“Multi-Emb”), multitask training of one task along with the natural protein task (“NP”), multitask training without the PSI-BLAST embedding module but initializing the amino acid embedding by using the natural protein task (“NP only”), multitask training including the natural protein task (“All3”), “All3” with Viterbi post-processing (“All3+Vit”) and a previously reported method (“Previous”). Each row corresponds to a single task. The 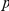-value column indicates whether the difference between “Single” and “All3+Vit” is significant, according to a Z-test. The “CV” column is computed based on the accuracies separately for each cross-validation fold. It counts the percentage of CV folds in which the “All3+Vit” method outperforms the “Single” method. Rows labeled “(prot)” or “(seg)” report the protein- or segment-level accuracy, rather than residue-level accuracy. For the “NP” setting, the “*” in the “Embedding?” row indicates that this network uses the pre-trained embedding layer from the natural protein task.

Thus far, these observations are only qualitative. However, to avoid problems with multiple testing correction, we chose not to perform a statistical test comparing the performance of each algorithm with and without the embedding layer. Instead, we perform at the end a statistical test jointly with respect to all three of our hypotheses.

### Multitask learning

Next, we evaluate the performance improvement to be gained by performing multitask learning when using just the PSI-BLAST features. Columns 1 and 3 (“Single” and “Multi”) in Table 2 compare the performance of networks trained one task at a time, versus networks trained in a multitask fashion. For the multitask learning, we experimented with various joint training schemes, and we settled upon the following. First, we pre-train a joint network for four “larger” tasks–dssp, ss, sar, saa. We then use the resulting learned joint model as a starting point for joint learning of nine tasks–the original four, plus dna, cc, ppi, sp and tm. For task cb513ss, to avoid overfitting between the ss and cb513ss tasks, we train a separate joint network from the task sar and saa, then multitask the joint model with cb513ss.

In all 10 cases, multitask learning improves the amino acid level accuracy. Not surprisingly, the performance difference is most dramatic for tasks with small training sets. For example, the performance on the DNA binding task, which has a data set of 693 proteins, jumps from 82.4% to 85.3%, and the prediction of coiled coil regions, with a data set of 765 proteins, improves from 88.6% to 93.1%. The secondary structure prediction tasks show how multitask learning helps with small data sets: for the small, CB513 data set, the accuracy improves by 3.7%, whereas for the larger secondary structure prediction benchmark, accuracy increases by 1.4%. Similar conclusions can be drawn by comparing the “Embed” and “Multi-Embed” columns: in all 10 cases, multitask learning improves the amino acid-level accuracy.

### Natural protein prediction

Finally, we evaluate the utility of the natural protein prediction task. The results in Table 2 confirm that multitasking with this natural protein task is an effective strategy to improve deep neural network training. Comparing column “Embed” to “NP”, we see that the performance improves in all cases. The benefit of the natural protein task is more apparent in conjunction with multitask learning, because the latter needs to handle much more complicated cases and to search in a larger parameter space, where a better starting position alleviates the difficulties associated with small training sets. Comparing the “Multi-Embed” column with “All3,” we see that adding the natural protein task improves the amino acid level accuracy for nine out of ten tasks. In general, however, adding the natural protein prediction task is not as beneficial as adding multitasking. This observation implies that inter-task dependencies provide more information than the contraints introduced via the natural protein embedding.

**Figure 4:**
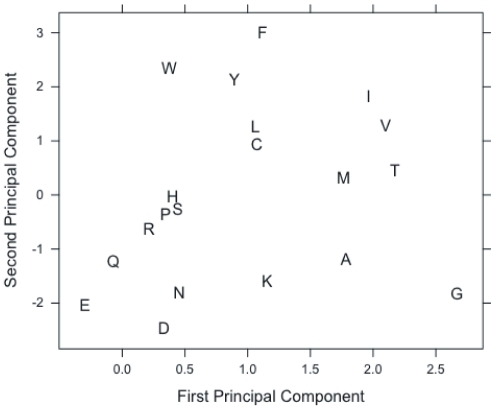
A learned amino acid embedding. The figure shows an approximation of a 15-dimensional embedding of amino acids, learned by a neural network trained on the natural protein task. The projection to 2D is accomplished via principal component analysis.

Furthermore, Figure 4 provides qualitative evidence that this embedding is helpful. Here, we used principal component analysis to project a learned, 15-dimensional amino acid embedding down to two dimensions for the purposes of visualization. The figure shows that amino acids with similar physical and chemical properties are embedded closely to one another. For instance, we observe clustered groups of hydrophilic (DEKQN) and hydrophobic (LMIVC) amino acids. We also observe that pairs of amino acids that are close in the embedding tend to have high BLOSUM62 scores [56], [57], indicating that they can readily substitute for one another in real protein sequences. Specifically, we calculate the 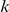-nearest neighbors for each amino acid, first based on our learned embedding and then based on BLOSUM62. We found that, with 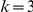, 62% of the amino acid neighbors identified by one method were also identified by the other. This result suggests that the learned embedding is closely related to BLOSUM62, even though it is learned purely from unlabeled protein sequences without any additional information.

To better understand the impact of the natural protein task, we also evaluated our system without the PSI-BLAST embedding module, but initializing the amino acid embedding with the embedding layer from the natural protein task. These results are reported in Table 2 in the column labeled “NP only”. The “NP only” network performs worse than “Embed” (which uses a randomly initialized amino acid embedding plus the PSI-BLAST embedding) in 9 out of 10 tasks; however, combining the natural language task with the PSI-BLAST embedding (i.e. “NP”) makes better predictions than “Single” in 8 out of 10 tasks. Thus, the embedding learned from the natural protein task is complementary to the PSI-BLAST features.

### Viterbi post-processing

Thus far, our neural network framework uses a labeling-per-amino-acid strategy without exploiting dependencies among the targeted classes. This approach assumes that the label of each position in a protein sequence can be predicted independent of nearby positions in the sequence. Empirically, this assumption fails dramatically for many local structure alphabets. For instance, the repeated label patterns of abcdefg in coiled coil predictions exhibit strong inter-label dependencies. It is straightforward to handle these local dependencies by applying a Viterbi (dynamic programming) post-processing step on the label posteriors. Adding this postprocessing step on our multitasking deep network ouputs (the “All3+Vit” column in Table 2) improves the the performance on 7 out of 10 of our tasks.

### Evaluation of the final system

The “All3+Vit” column in Table 2 represents the final performance of our multitask learning strategy. To evaluate the statistical significance of the difference between these results and the initial results provided by the single-task neural network, we performed a Z-test on each task. The result 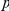-values are reported in Table 2. For almost all tasks, the “All3+Vit” setting is consistently better than the “Single” case, with most of the 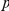-values smaller than 0.05. The only exception is the signal peptide task, assessed according to the protein level accuracy. The lack of statistical significance here is partly because the existing methods already achieve very good performance (99.5%) and partly because the protein-level metric provides fewer data points as input to the statistical test.

Relative to published, state-of-the-art prediction systems, our multitask, deep learning methodology fares quite well. For the secondary structure prediction task, our system achieved 80.3% amino acid level accuracy on the benchmark CB513 secondary structure prediction data set, which is slightly better than the state-of-the-art 80.0% [18]. For the signal peptide and transmembrane protein topology prediction tasks, our system outperforms Philius [26] on the same benchmark, though this improvement is partly because Philius does not make use of homology information. For prediction of coiled coil regions, our performance of 97.4% beats the best result (94%) on the same data set from [41] using the same evaluation setup. For the DNA binding task, our performance of 89.2% is slightly better than that of a previously described system [7].

### Comparison based on Precision/Recall/F1

As mentioned above, we compute precision, recall and F1 scores by treating each selected class as the positive class and all of the other classes as negatives for the multi-class tasks. The resulting comparison, provided in Table S1, shows that the conclusions based on accuracy in Table 2 still hold when we consider these alternative performance metrics. For example, we can see that most tasks' performance improves from “Single” to “Multi”, from “Multi” to “MultiEmbed”, from “MultiEmbed” to “All3” and from “All3” to “All3Vit”. For one task–protein-protein interaction–this trend is not maintained. In this case, multitasking does help (from “Single” to “Multi”), but adding the embedding and “natural protein” strategies make the predictions of the interaction (“P”) class much worse. This phenomenon may occur due to the small training set for this task, which could not provide enough examples for the more parameter-rich models like “MultiEmbed” and “All3”.

For the protein-protein interaction (“ppi”) task, the ISIS system [50] claimed its best performance as precision = 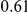 and recall = 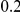 on the same data set as we use. The authors plotted a precision-recall curve (though the terms “accuracy” and “coverage” of interaction were used in [50]) for different cut-offs on the predicted score, and found this strongest prediction. In this paper, we use “0.5” as the universal cutoff for all the covered learning strategies to decide which class label an amino acid belongs to. Despite allowing ISIS to pick an optimal threshold and restricting our method to using a threshold of 0.5, under the “Multi” strategy, our system results in precision = 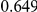 and recall = 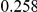, which is better than what the ISIS system [50] has reported.

## Discussion

We have described a multitask learning strategy for training a deep neural network architecture for the prediction of a variety of local protein properties. Our approach makes use of a learned embedding to share information across related tasks and uses the natural protein task to provide a good starting point for the learning of this embedding. We demonstrated that learning tasks simultaneously can improve generalization performance. In particular, when jointly trained with the natural protein task, our architecture achieved state-of-the-art performance in nearly all of the tasks that we considered.

We are not the first to suggest that multiple protein labeling tasks should be considered jointly. Many previous authors have combined tasks: transmembrane topology and signal peptide prediction [58], secondary structure and solvent accessibility [8], secondary structure, solvent accessibility and DNA-binding sites [7]. Nonetheless, we believe that the ability to train jointly on a large variety of tasks is novel and provides a flexible, robust prediction system that will be of great practical utility.

Our methodology could easily be adapted to additional tasks, such as the prediction of glycosylation sites, torsion angles, etc. The methodology could likely also benefit from further optimization. For example, it is possible to induce better pseudo-negative examples in the proposed natural protein task by adding biological knowledge, e.g., by simulating evolution. This, in turn, might increase our system's performance even more.

## Supporting Information

Table S1 The table lists, for each prediction task, the per-residue per-class performance, i.e. (precision, recall, F1, total number of provided positive amino acids, true positive) averaged per cross-validation test fold, achieved via single-task training of the neural network with just the PSI-BLAST features (“Single”), multitask training just using the PSI-BLAST features (“Multi”), multitask training including the amino acid embedding (“MultiEmbed”), multitask training including the natural protein task (“All3”), and multitask training including the natural protein task with Viterbi post-processing (“All3+Vit”).
(PDF)
