# Supplementary material for: A Unified Multitask Architecture for Predicting Local Protein Properties
Source: PLoS One. 2012 Mar 26;7(3):e32235. doi: 10.1371/journal.pone.0032235 (PMC3312883; doi:10.1371/journal.pone.0032235)
Supplement: Table S1 — The table lists, for each prediction task, the per-residue per-class performance, i.e. (precision, recall, F1, total number of provided positive amino acids, true positive) averaged per cross-validation test fold, achieved via single-task training of the neural network with just the PSI-BLAST features (“Single”), multitask training just using the PSI-BLAST features (“Multi”), multitask training including the amino acid embedding (“MultiEmbed”), multitask training including the natural protein task (“All3”), and multitask training including the natural protein task with Viterbi post-processing (“All3+Vit”). (PDF) [file pone.0032235.s001.pdf]

Table 1: **Comparison of learning strategies based on per-residual per-class precision / recall / F1**

Task	Strategy	Class	Precision	Recall	F1	ave Num. AA in test	Num. true positive
ssp	Single	H	67.63	82.56	74.35	185051.4	152800.2
ssp	Single	C	72.31	80.76	76.30	210064.4	169633.4
ssp	Single	E	51.08	69.87	59.01	108603.4	75889.2
ssp	Multi	H	69.41	83.89	75.96	185051.4	155246.0
ssp	Multi	C	74.10	81.84	77.77	210064.4	171906.6
ssp	Multi	E	53.55	72.12	61.46	108603.4	78317.8
ssp	MultiEmbed	H	70.30	85.00	76.95	185051.4	157310.0
ssp	MultiEmbed	C	75.22	82.25	78.58	210064.4	172770.0
ssp	MultiEmbed	E	54.99	73.14	62.78	108603.4	79424.8
ssp	All3	H	70.38	85.00	76.99	185051.4	157305.8
ssp	All3	C	75.25	82.34	78.64	210064.4	172969.0
ssp	All3	E	55.08	73.16	62.84	108603.4	79449.2
ssp	All3Vit	H	70.05	86.38	77.35	185051.4	159867.2
ssp	All3Vit	C	75.67	81.77	78.58	210064.4	171726.6
ssp	All3Vit	E	55.28	72.30	62.64	108603.4	78513.8
cb513	Single	H	62.44	76.62	68.75	4124.3	3167.9
cb513	Single	C	68.70	81.04	74.31	5116.4	4139.9
cb513	Single	E	48.29	65.99	55.64	2717.4	1793.0
cb513	Multi	H	66.43	83.13	73.83	4124.3	3428.0
cb513	Multi	C	73.62	82.12	77.63	5116.4	4198.3
cb513	Multi	E	54.34	70.30	61.28	2717.4	1910.9
cb513	MultiEmbed	H	66.97	83.61	74.35	4124.3	3449.7
cb513	MultiEmbed	C	74.10	82.57	78.09	5116.4	4221.0
cb513	MultiEmbed	E	55.10	70.60	61.88	2717.4	1919.1
cb513	All3	H	66.97	83.66	74.37	4124.3	3450.1
cb513	All3	C	74.15	82.49	78.08	5116.4	4217.1
cb513	All3	E	55.12	70.74	61.94	2717.4	1923.3
cb513	All3Vit	H	66.87	84.86	74.76	4124.3	3496.0
cb513	All3Vit	C	74.97	81.08	77.89	5116.4	4152.0
cb513	All3Vit	E	55.29	72.08	62.56	2717.4	1958.4
dssp	Single	H	48.96	89.22	63.23	167584.4	149522.0
dssp	Single	S	4.65	13.58	6.93	47652.6	6470.6
dssp	Single	T	14.83	43.78	22.15	56533.8	24738.0
dssp	Single	I	0.00	0.00	0.00	106.6	0.0
dssp	Single	G	0.86	7.79	1.54	17467.0	1360.4
dssp	Single	B	0.00	0.06	0.00	5696.8	3.6
dssp	Single	E	34.51	76.86	47.63	102906.6	79095.0
dssp	Single	L	33.41	64.88	44.11	105771.4	68624.4
dssp	Multi	H	50.29	90.05	64.54	167584.4	150919.4
dssp	Multi	S	6.74	19.33	9.99	47652.6	9206.2
dssp	Multi	T	16.09	45.88	23.82	56533.8	25944.0
dssp	Multi	I	0.00	0.00	0.00	106.6	0.0
dssp	Multi	G	1.39	12.15	2.50	17467.0	2119.0
dssp	Multi	B	0.04	1.14	0.08	5696.8	64.4
dssp	Multi	E	36.04	78.94	49.49	102906.6	81238.6
dssp	Multi	L	34.74	64.66	45.19	105771.4	68370.2
dssp	MultiEmbed	H	51.16	90.69	65.42	167584.4	151999.0
dssp	MultiEmbed	S	7.65	21.43	11.28	47652.6	10206.6
dssp	MultiEmbed	T	17.19	48.25	25.35	56533.8	27282.8
dssp	MultiEmbed	I	0.00	0.00	0.00	106.6	0.0
dssp	MultiEmbed	G	1.85	15.71	3.31	17467.0	2742.0
dssp	MultiEmbed	B	0.06	1.63	0.12	5696.8	92.6
dssp	MultiEmbed	E	36.99	79.75	50.53	102906.6	82070.4
dssp	MultiEmbed	L	35.71	64.90	46.07	105771.4	68629.6

continued on next page

Task	Strategy	Class	Precision	Recall	F1	ave Num. AA in test	Num. true positive
dssp	All3	H	51.17	90.75	65.44	167584.4	152086.4
dssp	All3	S	7.44	20.71	10.94	47652.6	9866.8
dssp	All3	T	17.32	48.78	25.56	56533.8	27585.4
dssp	All3	I	0.00	0.00	0.00	106.6	0.0
dssp	All3	G	1.84	15.59	3.28	17467.0	2721.8
dssp	All3	B	0.07	1.94	0.14	5696.8	110.0
dssp	All3	E	36.99	79.77	50.54	102906.6	82086.8
dssp	All3	L	35.71	64.86	46.06	105771.4	68589.8
dssp	All3Vit	H	51.17	90.75	65.44	167584.4	152086.4
dssp	All3Vit	S	7.44	20.71	10.94	47652.6	9866.8
dssp	All3Vit	T	17.32	48.78	25.56	56533.8	27585.4
dssp	All3Vit	I	0.00	0.00	0.00	106.6	0.0
dssp	All3Vit	G	1.84	15.59	3.28	17467.0	2721.8
dssp	All3Vit	B	0.07	1.94	0.14	5696.8	110.0
dssp	All3Vit	E	36.99	79.77	50.54	102906.6	82086.8
dssp	All3Vit	L	35.71	64.86	46.06	105771.4	68589.8
sar	Single	A	78.72	76.29	77.48	246487.0	188045.2
sar	Single	B	77.93	80.23	79.06	257232.2	206378.4
sar	Multi	A	80.41	76.06	78.15	246487.0	187491.0
sar	Multi	B	78.20	82.21	80.14	257232.2	211424.6
sar	MultiEmbed	A	81.60	79.02	80.27	246487.0	194783.0
sar	MultiEmbed	B	80.50	82.89	81.66	257232.2	213181.0
sar	All3	A	81.69	78.97	80.29	246487.0	194675.2
sar	All3	B	80.48	83.01	81.71	257232.2	213463.2
sar	All3Vit	A	81.05	79.98	80.51	246487.0	197143.4
sar	All3Vit	B	81.06	82.08	81.57	257232.2	211140.6
saa	Single	A	74.43	68.42	71.30	176611.8	120847.4
saa	Single	B	83.66	87.31	85.45	327107.4	285590.2
saa	Multi	A	76.86	68.40	72.38	176611.8	120807.2
saa	Multi	B	83.90	88.88	86.32	327107.4	290718.8
saa	MultiEmbed	A	77.81	70.34	73.88	176611.8	124224.4
saa	MultiEmbed	B	84.77	89.17	86.91	327107.4	291651.4
saa	All3	A	77.79	70.61	74.02	176611.8	124717.6
saa	All3	B	84.89	89.11	86.95	327107.4	291474.0
saa	All3Vit	A	77.79	70.61	74.02	176611.8	124718.0
saa	All3Vit	B	84.89	89.11	86.95	327107.4	291473.6
dna	Single	N	83.89	96.96	89.94	34400.7	33341.0
dna	Single	P	59.88	19.32	28.97	7954.0	1561.3
dna	Multi	N	87.36	95.74	91.35	34400.7	32940.7
dna	Multi	P	68.03	40.35	50.41	7954.0	3133.0
dna	MultiEmbed	N	88.81	96.05	92.27	34400.7	33023.3
dna	MultiEmbed	P	72.08	47.88	57.25	7954.0	3692.3
dna	All3	N	90.36	96.45	93.28	34400.7	33157.0
dna	All3	P	75.48	55.61	63.43	7954.0	4253.0
dna	All3Vit	N	90.01	95.72	92.75	34400.7	32919.7
dna	All3Vit	P	72.42	53.96	61.16	7954.0	4146.7
sp	Single	S	15.12	79.59	25.36	4265.1	3393.9
sp	Single	O	72.09	62.35	66.82	32578.9	20265.1
sp	Single	N	82.37	89.77	85.90	69015.8	62020.8
sp	Multi	S	17.66	82.34	29.03	4265.1	3512.3
sp	Multi	O	76.42	67.79	71.78	32578.9	22010.2
sp	Multi	N	84.74	91.20	87.83	69015.8	62998.9
sp	MultiEmbed	S	18.26	84.74	29.99	4265.1	3612.9
sp	MultiEmbed	O	76.75	68.56	72.34	32578.9	22282.8
sp	MultiEmbed	N	85.14	91.14	88.02	69015.8	62915.3
sp	All3	S	18.44	84.76	30.23	4265.1	3614.7
sp	All3	O	77.46	68.07	72.41	32578.9	22121.9
sp	All3	N	85.03	91.59	88.17	69015.8	63245.2

continued on next page

Task	Strategy	Class	Precision	Recall	F1	ave Num. AA in test	Num. true positive
sp	All3Vit	S	31.46	93.29	46.67	4265.1	3978.0
sp	All3Vit	O	92.13	77.48	83.47	32578.9	25381.8
sp	All3Vit	N	90.17	96.98	93.37	69015.8	66962.5
tm	Single	S	0.27	14.87	0.53	116.3	15.4
tm	Single	O	87.58	98.13	92.52	37847.2	37140.1
tm	Single	M	33.30	68.77	44.68	3455.8	2372.3
tm	Single	N	0.00	0.00	0.00	198.1	0.0
tm	Single	R	0.00	0.00	0.00	50.6	0.0
tm	Single	I	21.81	14.09	16.98	4410.0	615.6
tm	Multi	S	0.78	34.99	1.52	116.3	37.6
tm	Multi	O	89.44	98.07	93.54	37847.2	37122.0
tm	Multi	M	39.02	79.49	52.13	3455.8	2748.0
tm	Multi	N	0.00	0.00	0.00	198.1	0.0
tm	Multi	R	0.00	0.00	0.00	50.6	0.0
tm	Multi	I	37.25	24.75	29.53	4410.0	1093.7
tm	MultiEmbed	S	0.65	26.71	1.26	116.3	30.6
tm	MultiEmbed	O	89.75	98.12	93.73	37847.2	37142.7
tm	MultiEmbed	M	40.16	81.47	53.58	3455.8	2816.2
tm	MultiEmbed	N	0.00	0.00	0.00	198.1	0.0
tm	MultiEmbed	R	0.00	0.00	0.00	50.6	0.0
tm	MultiEmbed	I	39.70	26.60	31.64	4410.0	1177.5
tm	All3	S	0.71	28.50	1.39	116.3	33.9
tm	All3	O	89.82	98.21	93.80	37847.2	37172.2
tm	All3	M	40.56	81.55	53.93	3455.8	2818.1
tm	All3	N	0.00	0.00	0.00	198.1	0.0
tm	All3	R	0.00	0.00	0.00	50.6	0.0
tm	All3	I	40.86	27.28	32.47	4410.0	1201.3
tm	All3Vit	S	1.83	53.56	3.51	116.3	64.1
tm	All3Vit	O	93.12	97.69	95.31	37847.2	36977.4
tm	All3Vit	M	49.33	83.89	61.69	3455.8	2899.4
tm	All3Vit	N	0.30	7.16	0.57	198.1	10.0
tm	All3Vit	R	0.17	9.12	0.33	50.6	5.6
tm	All3Vit	I	55.38	55.44	53.76	4410.0	2484.2
coilcoil	Single	e	23.53	73.12	35.53	1918.1	1404.6
coilcoil	Single	c	23.39	72.34	35.29	1918.4	1392.4
coilcoil	Single	a	23.61	73.93	35.72	1912.9	1416.5
coilcoil	Single	b	23.60	73.90	35.71	1912.1	1416.6
coilcoil	Single	g	23.57	73.81	35.66	1909.7	1410.5
coilcoil	Single	N	89.19	95.12	92.04	31006.8	29475.7
coilcoil	Single	d	23.37	71.98	35.21	1921.0	1385.6
coilcoil	Single	f	23.77	74.75	36.00	1914.8	1433.3
coilcoil	Multi	e	37.13	83.75	51.25	1918.1	1612.2
coilcoil	Multi	c	37.13	83.71	51.24	1918.4	1612.3
coilcoil	Multi	a	37.10	84.09	51.28	1912.9	1615.0
coilcoil	Multi	b	37.08	84.05	51.26	1912.1	1614.8
coilcoil	Multi	g	37.11	84.40	51.34	1909.7	1618.2
coilcoil	Multi	N	93.39	96.90	95.11	31006.8	30069.5
coilcoil	Multi	d	37.18	83.75	51.28	1921.0	1614.6
coilcoil	Multi	f	37.15	84.29	51.37	1914.8	1618.5
coilcoil	MultiEmbed	e	42.13	86.26	56.37	1918.1	1660.1
coilcoil	MultiEmbed	c	42.10	86.07	56.32	1918.4	1657.5
coilcoil	MultiEmbed	a	42.02	86.10	56.25	1912.9	1653.7
coilcoil	MultiEmbed	b	42.07	86.78	56.45	1912.1	1665.6
coilcoil	MultiEmbed	g	42.03	86.45	56.33	1909.7	1656.2
coilcoil	MultiEmbed	N	94.35	97.50	95.90	31006.8	30249.5
coilcoil	MultiEmbed	d	42.15	86.02	56.34	1921.0	1656.9
coilcoil	MultiEmbed	f	42.16	87.21	56.61	1914.8	1675.0
coilcoil	All3	e	43.04	85.83	57.08	1918.1	1652.2

continued on next page

Task	Strategy	Class	Precision	Recall	F1	ave Num. AA in test	Num. true positive
coilcoil	All3	c	43.09	86.72	57.33	1918.4	1670.0
coilcoil	All3	a	43.01	86.50	57.20	1912.9	1661.1
coilcoil	All3	b	43.00	86.53	57.21	1912.1	1662.4
coilcoil	All3	g	42.98	86.43	57.16	1909.7	1657.1
coilcoil	All3	N	94.40	97.73	96.03	31006.8	30315.6
coilcoil	All3	d	43.12	86.46	57.29	1921.0	1667.1
coilcoil	All3	f	43.10	87.10	57.41	1914.8	1672.2
coilcoil	All3Vit	e	56.95	92.91	70.35	1918.1	1785.1
coilcoil	All3Vit	c	56.93	92.83	70.33	1918.4	1784.0
coilcoil	All3Vit	a	56.85	92.98	70.30	1912.9	1781.8
coilcoil	All3Vit	b	56.86	92.68	70.23	1912.1	1776.2
coilcoil	All3Vit	g	56.83	92.95	70.27	1909.7	1778.3
coilcoil	All3Vit	N	96.98	98.16	97.57	31006.8	30451.9
coilcoil	All3Vit	d	56.99	92.98	70.40	1921.0	1788.7
coilcoil	All3Vit	f	56.89	92.96	70.32	1914.8	1782.4
tppi	Single	N	73.59	100.00	84.78	46148.7	46146.3
tppi	Single	P	21.67	0.02	0.03	16743.3	4.3
tppi	Multi	N	78.66	97.47	86.96	46148.7	45099.0
tppi	Multi	P	64.92	25.80	35.27	16743.3	3278.0
tppi	MultiEmbed	N	73.66	98.82	84.38	46148.7	45692.3
tppi	MultiEmbed	P	32.38	1.54	2.68	16743.3	200.7
tppi	All3	N	74.68	98.71	84.98	46148.7	45637.3
tppi	All3	P	49.19	6.68	10.28	16743.3	872.7
tppi	All3Vit	N	76.57	97.42	85.52	46148.7	45150.0
tppi	All3Vit	P	41.55	15.69	18.54	16743.3	2037.7

The table lists, for each prediction task, the per-residue per-class performance, i.e. (precision, recall, F1, total number of provided positive amino acids, true positive) averaged per cross-validation test fold, achieved via single-task training of the neural network with just the PSI-BLAST features ("Single"), multitask training just using the PSI-BLAST features ("Multi"), multitask training including the amino acid embedding ("MultiEmbed"), multitask training including the natural protein task ("All3"), and multitask training including the natural protein task with Viterbi post-processing ("All3+Vit").
